# Abdominal trachelectomy in a case of cervical myoma as a fertility sparing surgery

**DOI:** 10.1093/jscr/rjac557

**Published:** 2022-12-07

**Authors:** Abdulrahim Gari, Balqes F Alrajhi

**Affiliations:** Department of Obstetrics and Gynecology, Faculty of Medicine, Umm Al-Qura University, Makkah 24243, Saudi Arabia; Department of Obstetrics and Gynecology, King Faisal Specialist Hospital and Research Center, Jeddah 23433, Saudi Arabia; MBBS, College of Medicine, King Saud University, Riyadh, Saudi Arabia

## Abstract

Cervical leiomyoma is considered a rare pathology with limited treatment options especially if preserving fertility is a concern. Traditional fertility-preserving surgery such as myomectomy has been the mainstay of management if it is possible (Ferrari F, Forte S, Valenti G, Ardighieri L, Barra F, Esposito V, et al. Current treatment options for cervical leiomyomas: a systematic review of literature. *Medicina (Kaunas)* 2021;57:1–15). Trachelectomy was described as fertility-preserving surgery in patients with early-stage cervical cancer. However, recent studies manage patients with trachelectomy for benign pathology and suggest that; it as an alternative option that otherwise will be treated as hysterectomy (Ferrari F, Forte S, Valenti G, Ardighieri L, Barra F, Esposito V, et al. Current treatment options for cervical leiomyomas: a systematic review of literature. *Medicina (Kaunas)* 2021;57:1–15). A 33-year-old female, Para 3, referred to a gynecology clinic with a history of heavy menstrual flow, pelvic pain and pressure symptoms. She is known case of fibroid uterus however after having done pelvic MRI with contrast it confirmed the diagnosis of cervical myoma, measured 10 cm × 10 cm, which is intraoperatively managed by trachelectomy as a fertility preservation surgery. The surgery was complicated by ureterovaginal fistula, which was managed with a DJ stent conservatively. Cervical myoma is a rare pathology, and trachelectomy should be considered as an option for a woman who wants to preserve her fertility. Complication and obstetrical outcome should be discussed with the patient; hence, more studies are needed to address the management of cervical myoma, surgical complications and outcome of this procedure, especially in a benign condition.

## INTRODUCTION

Uterine fibroid is considered the most common benign pelvic pathology in females during reproductive age however, it is rare to see cervical myoma which accounts for <1% of uterine fibroid presentation [1].

Treatment option for cervical myoma is hysterectomy for post-menopausal woman. However, treatment option with fertility-preserving procedures is challenging that include myomectomy, trachelectomy and uterine artery embolisation [1]. Here we present rare case of cervical myoma that was treated by trachelectomy to preserve her fertility.

## CASE PRESENTATION

A 33-year-old Saudi Female, Gravida 3 Para 3, medically and surgically free, was referred to gynecology clinic with a history of heavy menstrual flow, pelvic pain and pressure symptoms. The patient had an initial history and physical examination that revealed a huge myoma filling and enlargement of the cervix. Pelvic ultrasound and MRI, in contrast, showed a 10 × 10 cm cervical leiomyoma with normal uterine corpus. The patient was counseled for an abdominal trachelectomy. Before surgery, the pre-operative assessment was done, and she was prepared and consented for trachelectomy with the insertion of cerclage and possible hysterectomy. Surgical complications are discussed in detail, especially ureteric and bladder injury.

## OPERATIVE STEPS

**Figure 1:**
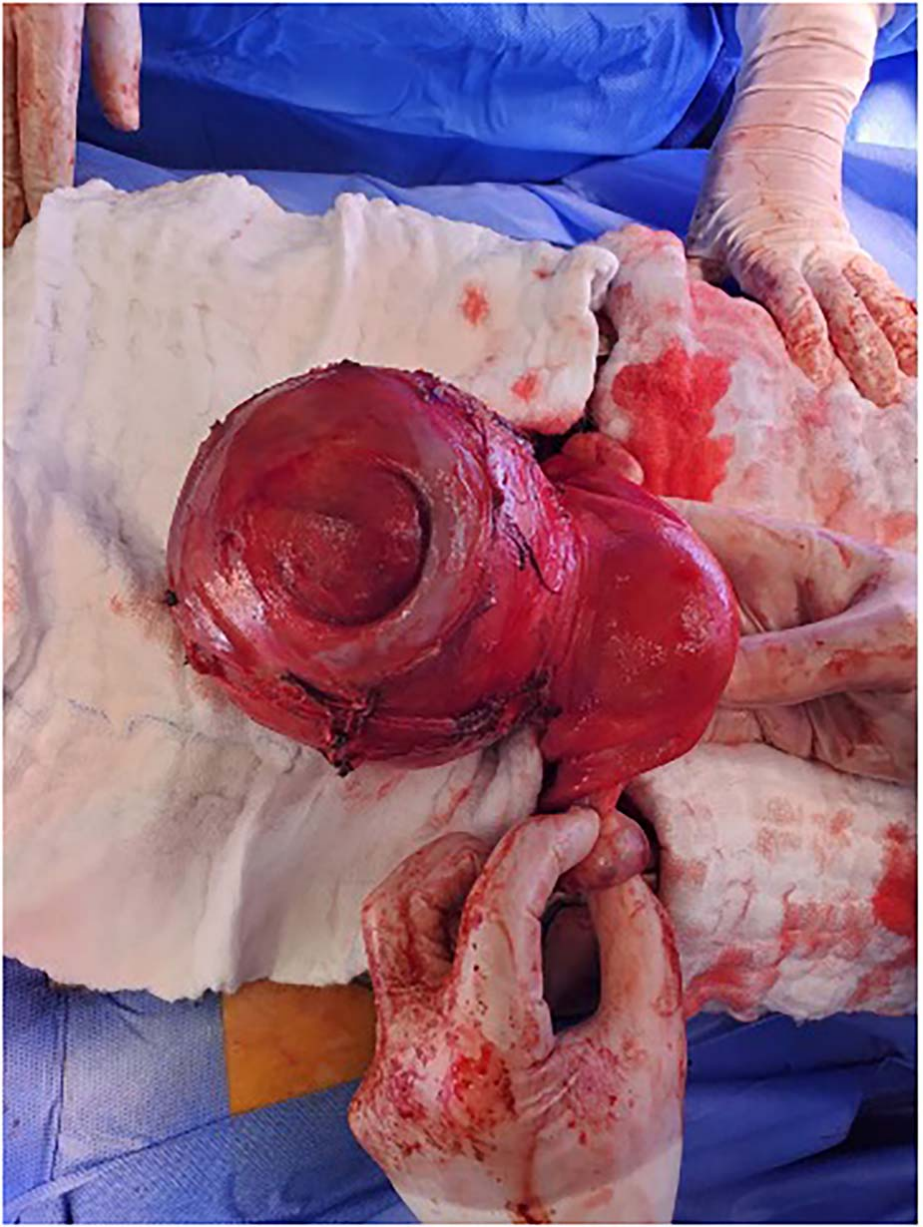
The cervical myoma attached to the uterus with intact infundibulopelvic ligament.

**Figure 2:**
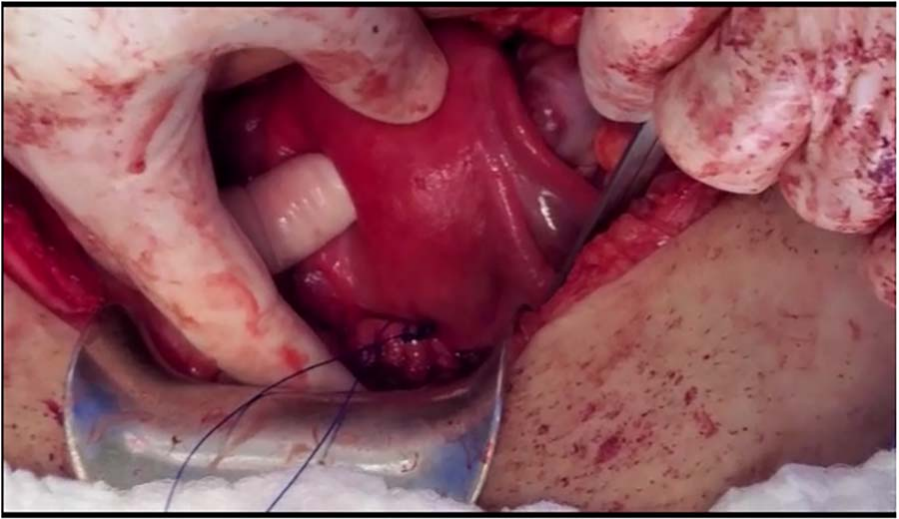
The cerclage placement after suturing the uterine isthmus to the vagina.

**Figure 3:**
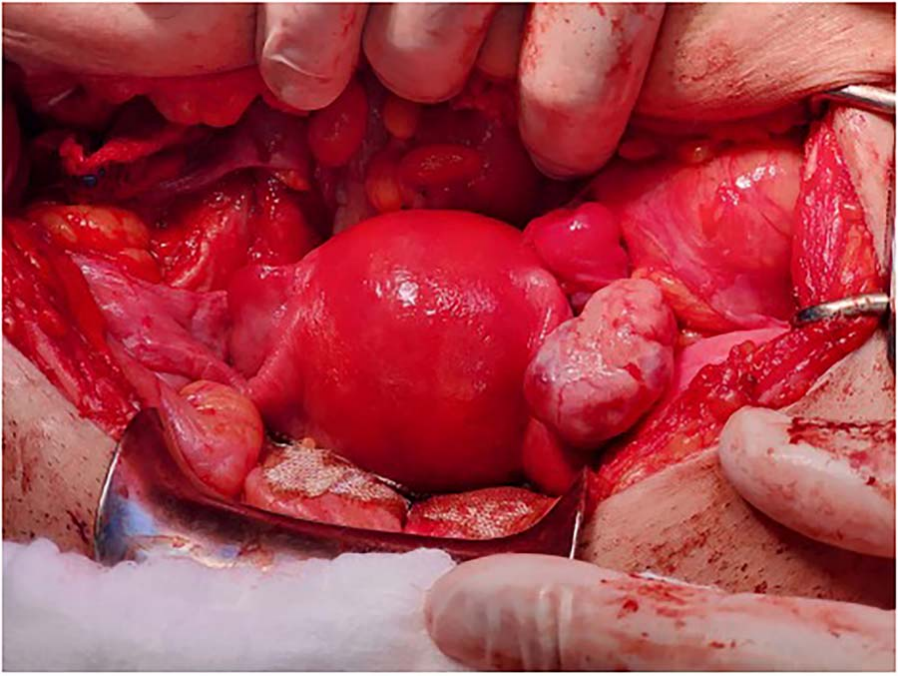
The final appearance of the uterus and ovaries.

Under spinal anesthesia, supine position, the patient underwent laparotomy with a transverse skin incision and midline incision of the fascia. Exploration confirmed the pre-operative diagnosis. The retroperitoneal space was entered, and ureters were identified bilaterally. A window was opened (broad ligament) at the level of the uterine artery to ligate uterine arteries using ligasure, ureterolysis was done and both ureters were skeletonised down to the ureteric tunnel (as in radical hysterectomy). The uterosacral ligament and cardinal ligament were ligated using ligasure, at the level of the cervico-vagina junction. An anterior colpotomy was done after the dissection of the bladder and creation of the bladder flap, and the anterior colpotomy was extended laterally from each side by using ligasure with careful attention to keep the ureters away. The circumcision of the cervix was completed by using Heaney clamps at the vaginal angles. The uterus was mobilised along with the cervix and the myoma and completely separated from the vagina (Fig. 1). At this point, cautery was used to amputate the cervix with careful attention to maintain the integrity of the infundibulo-pelvic ligament (Fig. 1). The uterus was sutured to the vagina with the box technique, where the posterior aspect of the vagina and cervix was sutured, followed by the lateral and anterior wall. After that, one proline permanent suture was used as a cerclage, used to support the uterus for future pregnancy (Figs 2 and 3).

## POST-OPERATIVE COURSE

**Figure 4:**
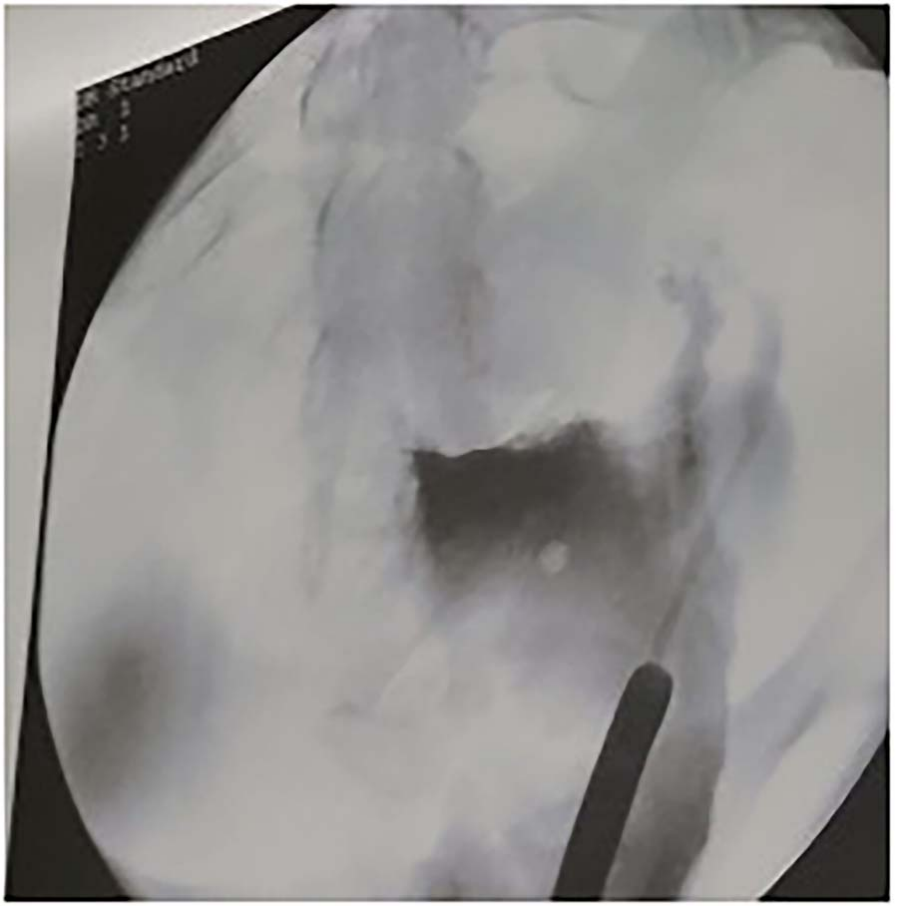
Leakage of contrast through the fistula.

**Figure 5:**
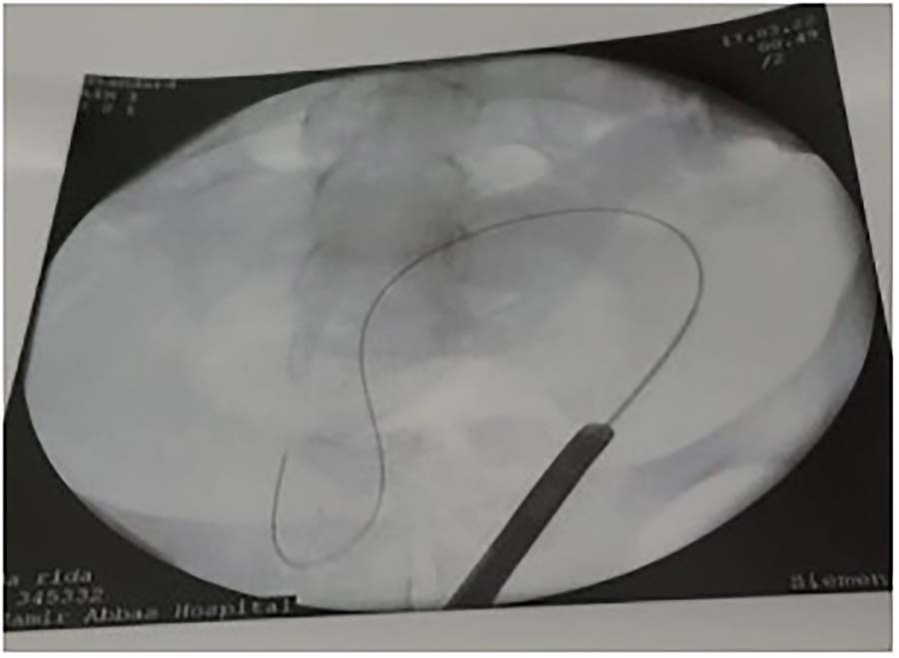
Guide wire passed through the UV fistula.

**Figure 6:**
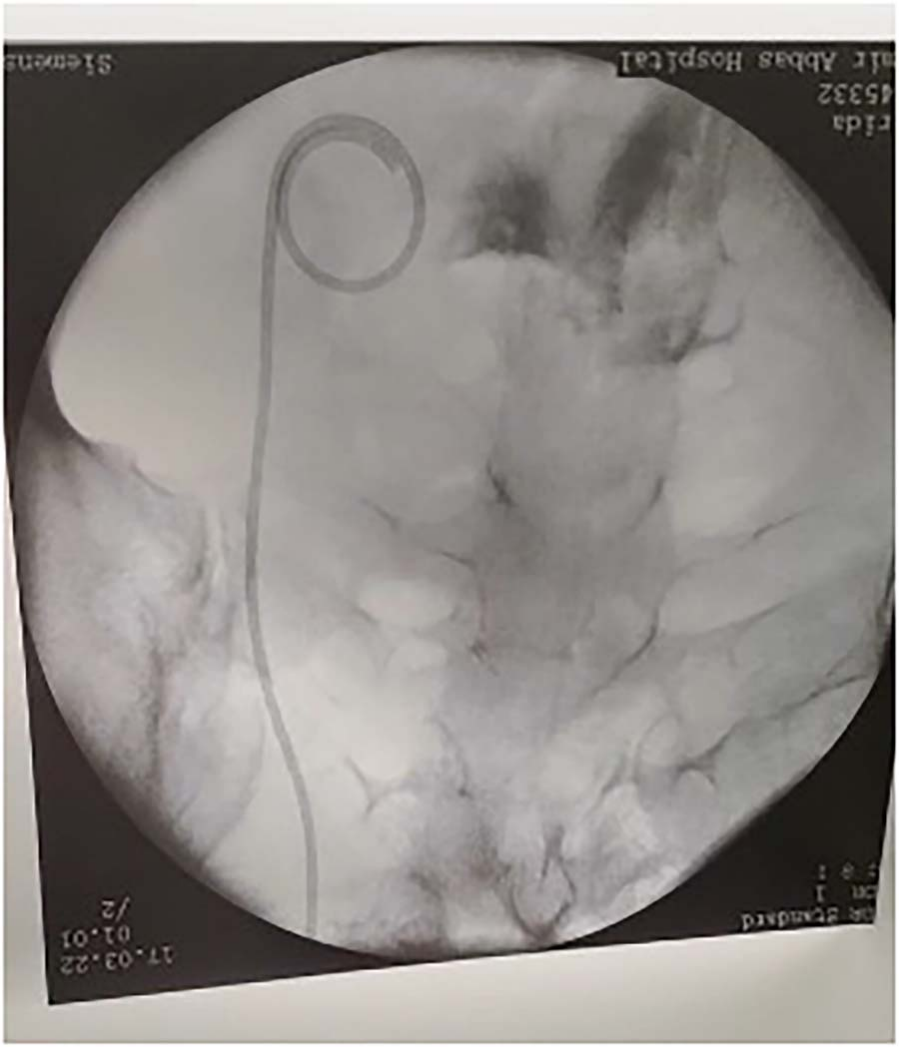
After insertion of DJ stent.

The patient tolerated the procedure very well and was transferred to the recovery room in stable condition. We did inform the patient that there is a small likelihood of developing a fistula within 2 weeks post-operatively. The patient did well in the post-operative period, recovered and was discharged to home in a stable condition. Unfortunately, on day 8, post-operatively, she started to have a leakage of urine per vagina. She was admitted for investigation, a CT Urogram was done with delayed images that confirmed the diagnosis of ureterovaginal fistula. The urologist was involved and consulted at this point; the plan was to take the patient for cystoscopy with retrograde ureteroscopy. Ureteroscopy wth retrograde fluoroscopy showed evidence of a ureterovaginal fistula (Figs 4 and 5) in which a DJ stent was inserted successfully (Fig. 6). The patient was explained about the post-operative course and plan. We decided to keep the stent for 12 weeks and then re-assess. The patient was covered by prophylaxis low-dose nitrofurantoin single daily dose of 100 mg. Fortunately, the leakage decreased gradually until it stopped. The patient was taken to the operating room to re-assess the fistula by cystoscopy and retrograde fluoroscopy. Retrograde fluoroscopy confirmed that the fistula healed. The stent was removed, and the patient was discharged to home in a stable condition.

Three months post-operatively, the patient was re-assessed clinically; she was doing fine, asymptomatic and had her menstrual period twice. Her period flow was average, not associated with dysmenorrhea, and there were no pressure symptoms. The final histology of the pelvic tumor was leiomyoma arising from the uterine cervix. There was no excess mitotic activity, nuclear atypia or necrosis.

## DISCUSSION

Cervical leiomyoma is considered a rare pathology; the classification of cervical leiomyoma depends on the location either an extra-cervical or intra-cervical type, with limited treatment options due to its rarity, which includes surgical, interventional radiology and conservative management [1]. Conservative management of symptomatic cervical myoma is preserved for special cases that are reported in literature such as pregnancy and refusal of patients [2]. A systematic review that studies treatment options for cervical myoma reveals that radiological management is a good option for a patient that opposes the surgery and wants to preserve the uterus. However, it shows a high rate of spontaneous abortion, cesarean section and malpresentation [1]. Laparoscopic myomectomy and hysterectomy have been the mainstay of treatment of cervical myoma [1]. However, generally, cervical myoma surgery is quite difficult due to the high risk of complications such as injury to adjacent organs, increased blood loss and poor operative field that needs an expert surgeon. Hysterectomy is the choice of surgery in the case of cervical myoma for post-menopausal patients [1]. However, cervical leiomyoma is prevalent mainly in the 25–40 y age group that prompts more fertility-preserving procedures like myomectomy, which is limited to less than 10-cm myoma, with the complications that involve possible hysterectomy, hemorrhage and risk of recurrence [3, 4]. Trachelectomy used to be an alternative treatment of early-stage cervical cancer as a fertility-preserving surgery [5]; there are two approaches to perform this surgery: either abdominal or laparoscopic without significant deference between the two approaches; however. hospital stay and blood loss is less in laparoscopic trachelectomy [5]. Moreover, many studies reported the obstetric outcome in this procedure range from a 25 to 60% success rate with the assistance of fertility treatment. However, multiple considerations should be taken into account, such as most patients delivered between 23 and 37 weeks by a cesarean section [6]. Additionally, pre-term labor and pre-mature rupture of membrane are reported complications that require a frequent follow-up [6]. Furthermore, there is a higher surgical complications such as infection, hemorrhage and ureteral/ladder injury, but it is worth mentioning that all pervious studies was done in patients with cervical cancer rather than benign disease [7]. Recent case series review trachelectomy for benign pathology with good pregnancy outcomes and no complications such as wound infection, fever and urinary retention; all patients have uneventful pregnancy after 1 year [8]. A case report resembles ours of a 36-year-old female, Gravida 1 para 1, medically and surgically free. She was diagnosed with a 3-month history of abdominal distention; she dined dysmenorrhea and menorrhagia. They found huge cervical myoma that measures 20 cm on MRI, which is managed by trachelectomy without any complication [3]. There is another case report of successful pregnancy following myomectomy and abdominal trachelectomy in infertile women, which was complicated by the threatened abortion at 21 weeks and managed by tocolysis and at 25 weeks diagnosed by pre-mature rupture of the membrane. The baby boy was delivered at 26 weeks and discharged after 6 months, weighting 4520 g [9].

## CONCLUSION

Cervical myoma is a rare pathology, and trachelectomy should be considered an option for a woman who wants to preserve her fertility. The complication and obstetric outcome should be discuses with the patient in details; hence, more studies are needed to assess the incidence of complications and outcome of this procedure, especially in benign conditions.

## CONFLICT OF INTEREST STATEMENT

None declared.

## FUNDING

Not applicable.

## AUTHORS’ CONTRIBUTIONS

A.G.: overall supervision of the research project, and reviewer of the manuscript.

A.G., B.F.A: review of literature, study design, progress report, final report and manuscript writing.

All authors contributed to read and approved the final manuscript.

## DATA AVAILABILITY

Not Applicable.

## References

[1] Ferrari F , Forte S, Valenti G, Ardighieri L, Barra F, Esposito V, et al. Current treatment options for cervical leiomyomas: a systematic review of literature. Medicina (Kaunas) 2021;57:1–15. 10.3390/medicina57020092 PMC7911900 33494297

[2] Keriakos R , Maher M. Management of cervical fibroid during the reproductive period. Case Rep Obstet Gynecol 2013;2013:1–3. 10.1155/2013/984030 PMC3787639 24109537

[3] Wong J , Tan GHC, Nadarajah R, Teo M. Novel management of a giant cervical myoma in a premenopausal patient. BMJ Case Rep 2017;2017:1–5. 10.1136/bcr-2017-221408 PMC5652390 28993358

[4] Abu Hashim H , Al Khiary M, El Rakhawy M. Laparotomic myomectomy for a huge cervical myoma in a young nulligravida woman: a case report and review of the literature. Int J Reprod Biomed 2020;18:135–44. 32259008 10.18502/ijrm.v18i2.6421 PMC7097170

[5] He Z , Bian C, Xie C. Fertility-sparing surgery in early-stage cervical cancer: laparoscopic versus abdominal radical trachelectomy. BMC Womens Health 2022;22:1–8. 35717185 10.1186/s12905-022-01826-7 PMC9206326

[6] Okugawa K , Kobayashi H, Sonoda K, Kaneki E, Kawano Y, Hidaka N, et al. Oncologic and obstetric outcomes and complications during pregnancy after fertility-sparing abdominal trachelectomy for cervical cancer: a retrospective review. Int J Clin Oncol 2017;22:340–6. 27804040 10.1007/s10147-016-1059-9

[7] Pareja R , Rendón GJ, Sanz-Lomana CM, Monzón O, Ramirez PT. Surgical, oncological, and obstetrical outcomes after abdominal radical trachelectomy—a systematic literature review. Gynecol Oncol 2013; 131:77–82. 23769758 10.1016/j.ygyno.2013.06.010

[8] Del Priore G , Klapper AS, Gurshumov E, Vargas MM, Ungar L, Smith JR. Rescue radical trachelectomy for preservation of fertility in benign disease. Fertil Steril 2010;94:1910.e5–7. 10.1016/j.fertnstert.2010.03.019 20416873

[9] Kamei Y , Miyoshi A, Wakui N, Hara T, Kanao S, Naoi H, et al. Successful pregnancy following myomectomy accompanied with abdominal radical Trachelectomy for an infertile woman with early cervical cancer: a case report and literature review. Case Rep Surg 2018;2018:1–4. 10.1155/2018/5623717 PMC6051115 30057847

